# Supplementary material for: Language, economic and gender disparities widen the scientific productivity gap
Source: PLoS Biol. 2025 Sep 18;23(9):e3003372. doi: 10.1371/journal.pbio.3003372 (PMC12445530; doi:10.1371/journal.pbio.3003372)
Supplement: S1 Table — (DOCX) [file pbio.3003372.s001.docx]

**S1 Table**. Number of survey participants by English proficiency, income level, and gender identity.

| English proficiency | Income level | Gender | Number of participants |
| --- | --- | --- | --- |
| English native | High | Female | 58 |
|  |  | Male | 53 |
|  |  | Other | 2 |
|  | Lower-middle | Female | 12 |
|  |  | Male | 29 |
|  |  | Other | 0 |
| Moderate | High | Female | 40 |
|  |  | Male | 68 |
|  |  | Other | 0 |
|  | Lower-middle | Female | 92 |
|  |  | Male | 71 |
|  |  | Other | 4 |
| Low | High | Female | 84 |
|  |  | Male | 207 |
|  |  | Other | 2 |
|  | Lower-middle | Female | 53 |
|  |  | Male | 128 |
|  |  | Other | 5 |
