# Supplementary material for: Language, economic and gender disparities widen the scientific productivity gap
Source: PLoS Biol. 2025 Sep 18;23(9):e3003372. doi: 10.1371/journal.pbio.3003372 (PMC12445530; doi:10.1371/journal.pbio.3003372)
Supplement: S2 Table — Survey participants whose first language is English were excluded from this analysis. Number of years in research was centered before the analysis. The reference category for English proficiency, Income level, Gender, and Discipline was Low English proficiency, High income, Male, and Conservation biology, respectively. Significant results are shown in bold. The bias-adjusted estimate of mean-square error (used as a predictive measure) based on the 10-fold cross-validation of the final model is 240.55, representing a 24.26% improvement from the null model with 317.61. (DOCX) [file pbio.3003372.s002.docx]

**S2 Table**. Results of a generalised linear model (with a negative binomial distribution) of factors explaining variations in the number of English-language peer-reviewed papers published by survey participants whose first language is not English (n = 754). Survey participants whose first language is English were excluded from this analysis. Number of years in research was centred before the analysis. The reference category for English proficiency, Income level, Gender, and Discipline was Low English proficiency, High income, Male, and Conservation biology, respectively. Significant results are shown in bold. The bias-adjusted estimate of mean-square error (used as a predictive measure) based on the 10-fold cross-validation of the final model is 240.55, a 24.26% improvement from the null model with 317.61.

| Coefficients | Estimate | Standard error | z | p |
| --- | --- | --- | --- | --- |
| Intercept | 2.16 | 0.090 | 23.98 |  |
| **Number of years in research** | **0.052** | **0.0043** | **12.06** | **< 0.20 × 10^-15^** |
| English proficiency – moderate | -0.011 | 0.072 | -0.15 | 0.88 |
| **Number of years in English-speaking countries** | **0.056** | **0.010** | **5.58** | **2.45 × 10^-8^** |
| **Income level** – **lower-middle** | **-0.51** | **0.074** | **-6.85** | **7.64 × 10^-12^** |
| Gender – other | -0.079 | 0.29 | -0.27 | 0.78 |
| **Gender** – **female** | **-0.46** | **0.070** | **-6.51** | **7.72 × 10^-11^** |
| **Discipline** – **ecology** | **0.21** | **0.091** | **2.35** | **0.019** |
| Discipline – evolutionary biology | 0.17 | 0.12 | 1.47 | 0.14 |
| Discipline – other | 0.22 | 0.13 | 1.73 | 0.084 |
| **Discipline** – **other biological sciences** | **0.30** | **0.11** | **2.70** | **0.0070** |
| Number of years in research × English proficiency – moderate | 0.0047 | 0.0061 | 0.78 | 0.44 |
| Number of years in research × Gender – other | -0.044 | 0.023 | -1.93 | 0.054 |
| Number of years in research × Gender – female | 0.012 | 0.0071 | 1.66 | 0.097 |
