# Supplementary material for: Language, economic and gender disparities widen the scientific productivity gap
Source: PLoS Biol. 2025 Sep 18;23(9):e3003372. doi: 10.1371/journal.pbio.3003372 (PMC12445530; doi:10.1371/journal.pbio.3003372)
Supplement: Attachment — Submitted filename: Responses_Amano.docx [file pbio.3003372.s006.docx]

**Response to the reviewers for "Language, economic, and gender disparities widen the scientific productivity gap " (PBIOLOGY-D-25-00053R1)**

---------------------------------------------------------------------------------------------------------------------------

**In response to Editor’s comments**

(Line numbers are those for the file with track changes)

Comment #1

You'll see that reviewer #1 is positive, and has a number of points that can be addressed textually (clarifications, limitations). Reviewer #2 is also very positive, and has a few suggestions, some of which might involve further analysis (e.g. country as fixed effect). Reviewer #3 is more cautious, and wants you to consider a number of alternative explanations and limitations, plus several analyses (with some overlap with rev #2), including the use of alternative metrics of “success.”

Thank you very much for your positive evaluation. We have accepted or addressed the suggestions provided by the three reviewers, and made corrections accordingly.

In particular, we have expanded the section on limitations in the Discussion (L254-277), where we explained the possibility that the results may suffer from survivorship bias, the importance of using other metrics of scientific productivity (both to address Reviewer #3’s comment), and the role of other factors, especially individual-level factors, in explaining the variation in scientific productivity (to address Reviewers #2 and #3’s comment). We have also conducted 10-fold cross validation to assess the predictive performance of the final models compared to the null model, following Reviewer #2’s suggestion.

There are, nevertheless, a few comments where, after careful thought, we have decided to maintain the original version. Each of these points is listed in our responses to the reviewers with a full explanation of our reasoning. We hope that the Editor will find our responses satisfactory, but should any concerns remain, we will be more than happy to discuss further.

Regarding PLOS' data policy, as we discussed when publishing our previous paper (<https://doi.org/10.1371/journal.pbio.3002184>), our ethics approval prevents us from sharing raw data to secure confidentiality of the respondents. We therefore followed the approach used in our previous paper and provided all values plotted in Figs. 1 and 2—except for the raw data points in Fig. 1—as S1 Data. We have explicitly explained this in **Data and materials availability** (L454-461).

We have also provided all figures now as TIFF and all supplementary tables as separate files with their legends listed after References in the main text.

**In response to Reviewer #1’s comments**

(Line numbers are those for the file with track changes)

Comment #1

I enjoyed reading this manuscript. The topic is very relevant and timely, as it is becoming clear that the current evaluation system in science is not promoting diversity. This study uses a survey approach to quantify the gender, language, and economic correlates of scientific productivity measured using the most common measure of productivity - scientific publications.

Thank you so much for this positive evaluation. We have carefully considered all the comments by Reviewer #1 and revised the manuscript accordingly. We hope that Reviewer #1 will find our responses satisfactory, but should any concerns remain, we will be more than happy to discuss further.

Comment #2

I only have a few comments:

1. I am not sure if the wording 'to their full potential' is meaningful. What would a full potential be? Would it be potential that one would reach if one would just do science without any restrictions? I think rather than referencing one's work in respect to some full potential standard, it needs to be highlighted that the output of researchers is different, and this is depended on many factors, some of which cannot be changed. And thus the person has reached their full potential given those circumstances. My full potential is inevitably different than a full potential of someone else, and I cannot change it, but those who evaluate me can take the circumstances into account when evaluating me. Or take different metrics.

This is a good point. We have reconsidered the wording and now replaced or deleted it as follows:

Lines 33: From “prevent scientists from reaching their full potential” to “prevent people with diverse backgrounds from contributing to and participating in science”

Line 47: From “contribute to science to their full potential” to “contribute to science in an equal manner”

Line 233: Removed

Comment #3

2. Line 65 - unclear meaning of 'apparent'.

We initially used “apparent scientific productivity” in this paragraph because most metrics, such as the number of English-language papers published, do not necessarily indicate the “true” scientific productivity (e.g., the sum of English-language and non-English-language papers would better indicate one’s true scientific productivity for non-native English speakers, as is shown in this study). But we agree that its meaning is a little unclear here, so we have decided to remove “apparent” in Lines 69 and 78.

Comment #4

3. I suggest adding some basic summary info on the survey at the beginning of results, such as number of responses per country etc. This would facilitate putting the results into context.

The number of responses per country was shown in Lines 353-354, but following the suggestion by Reviewer #1, we have also included the information at the beginning of results (Lines 92-96).

Comment #5

4. Lines 88-90 - it is unclear if this sentence compares woman vs man, non-native vs native speakers etc, or all the former group vs all of the latter group.

This sentence means that women publish less than men, non-native English speakers publish less than native English speakers, and those form lower income countries publish less than those from higher income countries. We believe the word “respectively” indicates that this is a pair-wise comparison and we prefer the original concise sentence, but if Reviewer #1 had a specific phrasing in mind, we will be happy to consider it.

We have realised that our original sentence describing the results in Abstract was not clear, and therefore have revised it as follows (L38-41):

From “Being a non-native English speaker from a low-income country is associated with a further 25% reduction.” to “Being a woman, a non-native English speaker, and from a low-income country is associated with up to a 70% reduction, compared to male native English speakers from a high-income country.”

Comment #6

5. Unclear meaning of the sentence lines 98-99

This indicates that there was no significant interacting effect between the number of years in research and income level. For increased clarity we have added an explanation in L107-108.

Comment #7

6. Discussion currently lists one reasons why this survey might have underestimated the productivity gap. However, if I read the Methods correctly, I see two more reasons. First, the survey was in English (is this correct?), so probably responded by people who speak English. Those that do not speak English have likely not answer it. Second, emails were sent to only those that have first authored at least one publication in English, which means that those that do not have such authorship were excluded, which is also likely more those from underrepresented groups.

The survey was actually translated into the relevant languages for each nationality. We have realised that we did not explain this in the previous version and have now added it in L340-342 – thank you for pointing this out. Therefore the first point raised here should not be a problem.

We have indeed only included those who have at least one first-authored peer-reviewed paper in English, which, as Reviewer #1 points out here, can certainly cause an underestimation of the productivity gap. We have now included this in L256-258 and would like to thank Reviewer #1 for this useful suggestion.

Comment #8

7. Given that first and last authorship are usually more prestigious, do you think future surveys should also ask question about the authorship position?

We fully agree – we have included this in L266-268.

Comment #9

8. Methods - please specify if the survey was sent in English only.

Thanks for pointing this out – it was translated into relevant languages, which has now been clarified in L340-342.

**In response to Reviewer #2’s comments**

(Line numbers are those for the file with track changes)

Comment #1

Amano et al. perform an impressive cross-country survey of 908 environmental scientists to untangle the relative importance of linguistic, economic, and gender backgrounds on scientific productivity. I recommend this manuscript for publication with a few minor suggestions for improvement.

Thank you so much for this positive evaluation. We have done our best to address the points raised by Reviewer #2. Please see below our detailed response to each point.

Comment #2

1. The authors left out several crucial variables on the survey that would have clarified the results immensely. Both the job title of the respondent and the prestige of their workplace institution could have explained several of the effects from the paper. For instance, it can be that women are systematically excluded from tenure-track positions, where productivity can be higher due to running a lab. Or, women are excluded from elite institutions, where productivity is higher due to greater administrative support and smaller teaching load. These possible explanations should be included in the discussion.

Thank you for raising this important point. We agree that there are many other variables that were not collected in the survey (including job titles and institutions: please see the questionnaire survey here: https://doi.org/10.1371/journal.pbio.3002184.s028) but can potentially explain the scientific productivity of individual researchers. We have now included this in L273-274. We have also discussed the need to conduct longitudinal research and qualitative causal inference methods, such as process tracing and general elimination methodology, to investigate what we couldn’t do in our survey as future priorities (L260-266).

Comment #3

2. The authors group together all of the countries within income brackets together in the regression analysis.I would recommend controlling for each country specifically as a fixed effect, to isolate the effects within countries. This would strengthen the gender analysis, since none of the hypothesized mechanisms about gender depend on the country.

Thank you for this suggestion. Unfortunately, the design of the survey prevented us from controlling for country as suggested here. As we have only one or two countries for each combination of English proficiency and income level (see L332-336), the three variables (nationalities, English proficiency level, and income level) are almost perfectly collinear with each other. For example, using Nationality as a random factor in a negative binomial GLMM (which we would prefer, compared to including Nationality as a fixed effect) has indeed returned an error, pointing out a convergence issue. An attempt to calculate variance inflation factor to test multicollinearity has also indicated that there is perfect collinearity. We have explained this in L368-370.

Comment #4

3. In addition to inferential statistics such as the p-values and coefficient estimates, the authors should report predictive statistics. For instance, in performing (say) 10-fold cross-validation, how well does the model do at predicting a researcher's productivity, compared to an average baseline where everyone is predicted to produce the same number of papers.

Thank you for this helpful suggestion. Following this suggestion, we have now conducted 10-fold cross-validation and compared bias-adjusted estimates of mean-square error (MSE) between the final and null models (reported in the legends of Tables 1 and 2, and S2 Table, and explained in L386-389). Improvements in MSE were 13-24%, which was meaningful yet not necessarily high, potentially showing the importance of other factors, such as individual-level factors. We have discussed this point in L269-270.

**In response to Reviewer #3’s comments**

(Line numbers are those for the file with track changes)

Comment #1

The manuscript tried to address an important topic—structural disparities in scientific productivity related to language, income, and gender. The authors present a well-organized analysis using survey data and regression analysis. This study analyzes survey data from 908 environmental scientists across eight countries to examine how language, economic, and gender disparities relate to scientific productivity. The authors find that non-native English speakers, scientists from lower-income countries, and women report significantly fewer peer-reviewed publications compared to their native English-speaking, high-income country, and male counterparts. The authors argue that these disparities reflect structural inequities rather than differences in researcher ability or effort.

However, there are several key limitations before publication in a high-impact venue like PLOS Biology. The question I have about the manuscript are quite basic.

Thank you very much for your time to review our manuscript. We have done our best to address the issues raised by Reviewer #3. Please see below our responses to each comment and we will be more than happy to revise the manuscript if Reviewer #3 still finds any of our responses unsatisfactory.

Comment #2

First, the survey is limited to 908 environmental scientists from eight countries, and eligibility required authors to have at least one first-authored English-language publication. This introduces selection bias by excluding those who may have already exited academia due to systemic barriers—potentially underestimating disparities. The paper acknowledges this but does not sufficiently explore the implications for generalizability or suggest methods to address survivor bias in future research.

This is an important point that we are fully aware of, and as Reviewer #3 also mentioned here, we had already explained the issue in one of the paragraphs in Discussion (L258-260) and in Methods (L347-349). For increased clarity, we have now explicitly mentioned that our data collection potentially caused survivorship bias in the results (L260). We have also suggested approaches for addressing this issue, i.e., conducting longitudinal research to understand the differences in career trajectories between researchers with different backgrounds (L261-263), and qualitative causal inference methods, such as process tracing and general elimination methodology, to identify the mechanisms underlying the patterns found in this study (L263-266). However, addressing the survivorship bias would only widen the productivity gap we found in this study, and therefore, we still believe that our findings are sufficiently novel and worthy of publication.

Comment #3

Second, the study uses country-level proxies for both income and English proficiency rather than individual-level metrics. While practical, this potentially masks important within-country heterogeneity. For example, defining non-native English speakers as those from countries with "low" proficiency may oversimplify nuanced linguistic experiences. Furthermore, individual characteristics are widely known to be correlated with research capabilities. In addition, it is also worth considering including country fixed effect.

Thank you for this suggestion. We certainly agree that within-country heterogeneity caused by individual metrics is important. For this very reason, our original manuscript included a whole paragraph (L153-164) that explained the potential importance of individual English proficiency levels, and we had also conducted an analysis (shown in S1 Table), where we tested for a measure of individuals’ levels of English proficiency (the number of years spent living in countries where English is the first language) and showed that it indeed explained the variation in scientific productivity (see L159-164).

Of course, many other individual-level variables could explain the level of scientific productivity, but our survey unfortunately did not collect any other usable variable, and therefore we had noted the importance of more detailed variables in L268-272. For increased clarity, we have now added some examples of such individual-level variables (job titles and institutions) in L273-274.

Further, unfortunately, the design of the survey prevented us from controlling for country in the analyses. As we have only one or two countries for each combination of English proficiency and income level (see L332-336), the three variables (nationalities, English proficiency level, and income level) are almost perfectly collinear with each other. For example, using Nationality as a random factor in a negative binomial GLMM (which we would prefer, compared to including it as a fixed effect) has indeed returned an error, pointing out a convergence issue. An attempt to calculate variance inflation factor to test multicollinearity has also indicated that there is perfect collinearity. We have explained this in L368-370.

Comment #4

Thirdly, although the authors caution against making causal claims, some interpretations appear causal in tone. Furthermore, the mechanisms driving the observed productivity gaps are not deeply explored. Are disparities due to time costs, access to networks, publishing fees, mentorship, or other structural issues? Even speculative discussion or supporting evidence from qualitative research would enrich the paper's explanatory value.

When reporting the results, we tried to avoid making causal claims by e.g. not using “cause”, “lead to”, “result in” etc. But if Reviewer #3 strongly believes that there are specific expressions that are casual and should be rephrased, please let us know and we will be more than happy to consider it.

For the mechanisms driving the observed productivity gaps, we had cited multiple references showing numerous barriers for women, non-binary people, non-native English speakers and those from lower income countries in L55-67 and 169. But for increased clarity, we have now provided one example study that quantified such barriers for non-native English speakers (L169-173). We also proposed future studies to use qualitative causal inference methods (e.g., process tracing and general elimination methodology) to better explain the reasons behind patterns described in our results (L263-266).

Comment #5

Fourth, the regression results are not presented in a professional or standardized format. I recommend referring to classical economics papers as a guide for reporting. For example, with respect to the gender variable, the male category appears to be the omitted baseline, but this should be clearly stated in the table notes or main text. Additionally, while there are five discipline categories, four coefficients are shown. It seems the omitted reference category is missing from the presentation and should be explicitly identified. Most importantly, a more straightforward and interpretable way to demonstrate the effects of gender, language, and income would be to visualize their predicted margins, after controlling for all other covariates in the model. This would provide a clearer sense of the magnitude and direction of these effects, making the results more accessible to readers.

In the legend of each table, we had already explained the reference category for each categorical variable, for example in Table 1 as “The reference category for English proficiency, Income level, Gender, and Discipline was English native, High income, Male, and Conservation biology, respectively.” (L136-137). Same for Table 2 and S2 Table. Only four coefficients for Discipline are shown in those tables because one of the disciplines (Conservation biology) was used as the reference category, and hence its coefficient was zero. This is clearly described in each legend.

Predictive margins are defined for example in the Stata manual (https://www.stata.com/manuals/rmargins.pdf) as “*a statistic based on a fitted model calculated over a dataset in which some of or all the covariates are fixed at values different from what they really are. For instance, after a linear regression fit on males and females, the marginal mean (margin of mean) for males is the predicted mean of the dependent variable, where every observation is treated as if it represents a male*”. We believe that this is what we had already shown in Fig. 2, where we estimated, for example, the expected number of English-language papers for male and female researchers, with varying numbers of years in research, while the other variables are fixed (i.e., assuming a native English speaker in conservation biology from a high-income country), and calculated the absolute and percentage differences between male and female researchers to visualise the gender effect (the pink vs orange lines in Fig.2; please also see L390-416 for the full method explanation). We had done the same to help visualise the linguistic effect (comparing native vs non-native English speakers while the other variables were fixed) and the economic effect (comparing researchers from a high- vs lower-middle income countries while the other variables were fixed). We prefer showing the absolute and percentage differences, rather than the predictive margins themselves, as it would then be easier to quantify and interpret the gender, linguistic, and economic disparities.

Comment #6

Fifth, the number of publications is used as the sole metric to describe scientists' productivity. However, this is a rather limited measure. More meaningful indicators of a scientist's success should be considered—such as citation counts, number of papers published in top field journals, and other quality-based metrics. Incorporating these measures would provide a more robust and nuanced evaluation of scientific productivity.

We fully agree that the number of publications is one of the many metrics that describe scientists’ productivity; however, unfortunately, we did not collect any other metric in the survey and therefore cannot test their association with individuals’ gender, linguistic, and economic backgrounds. We have therefore added an explanation that future studies should also look at those metrics (L266-268).

Comment #7

Finally, the focus on environmental sciences and the geographic distribution of respondents (e.g., heavy representation from Japan) may skew findings. The authors should discuss the limits of disciplinary generalizability and whether similar patterns would hold in fields with different publishing cultures or collaboration norms.

Thank you for this suggestion – we have incorporated the following discussion in L274-277:

“The survey data used in this study are limited only to researchers in environmental sciences and within the eight nationalities. We call for further research to assess whether similar patterns would hold in other disciplines and countries with different publishing cultures and collaboration norms”.

Comment #8

The paper concludes with important implications for research assessment but lacks actionable policy suggestions. I hope these comments are helpful in the development of your manuscript.

Based on our findings, our original manuscript had proposed two actionable policy suggestions: (i) “*being a non-native English speaker and from a lower income country also should be a factor that is considered explicitly in any research assessment as a major impediment to the research capacity, productivity and contribution of scientists.*” (L292-295) and (ii) “*Including non-English-language publications in research assessment by individuals, institutions, and funders*” (L301-303). For increased clarity, we have included the part in red in the second suggestion. These two suggestions were also included in the abstract: “*We call for an explicit effort to consider linguistic, economic, and gender backgrounds and incorporate non-English-language publications when assessing the performance and contribution of scientists*” (L42-44). We believe that these are all important, novel and actionable suggestions for research assessment at any level, but we will be more than happy to consider revising any part of this if Reviewer #3 still thinks that it is unclear.
